# Diagnostic accuracy of [^99m^Tc]Tc-Sestamibi in the assessment of thyroid nodules

**DOI:** 10.18632/oncotarget.21866

**Published:** 2017-10-17

**Authors:** Anna Yordanova, Soha Mahjoob, Philipp Lingohr, Jörg Kalff, Andreas Türler, Holger Palmedo, Hans-Jürgen Biersack, Glen Kristiansen, Jamshid Farahati, Markus Essler, Hojjat Ahmadzadehfar

**Affiliations:** ^1^ Department of Nuclear Medicine, University Hospital Bonn, Bonn, Germany; ^2^ Department of Surgery, University Hospital Bonn, Bonn, Germany; ^3^ Department of General and Visceral Surgery, Johanniter-Krankenhaus Bonn, Bonn, Germany; ^4^ Institute of Radiology and Nuclear Medicine, PET-CT Center, Bonn, Germany; ^5^ Institute of Pathology, University Hospital Bonn, Bonn, Germany; ^6^ Department of Nuclear Medicine, Bethesda Hospital, Duisburg, Germany

**Keywords:** sestamibi, MIBI, thyroid nodules, thyroid cancer, cold nodule

## Abstract

[^99m^Tc]Tc-Sestamibi (MIBI) is an increasingly used tool for evaluation of thyroid nodules. However, there is a lack of evidence about the accuracy of this method in the European population. The aim of this study was to assess the utility of MIBI for the differentiation of thyroid nodules in a large cohort. 161 patients underwent MIBI, followed by a thyroidectomy. We used a dual phase MIBI protocol. Interpretation of the images included a scoring system from 0 (absent) to 3 (increased); this was to provide a scale for the uptake of the thyroid nodule in comparison to the paranodular tissue. Additionally, we evaluated the tracer uptake trend in late images compared to early images. We used the final histopathology as the reference standard. Scores 0-1 in early images, scores 0-2 in late images, and an absence of increasing uptake in the thyroid nodule in late images, showed the best predictive values to exclude malignancy, respectively (negative predictive value (NPV) 89%). Highest sensitivity (91%) for malignant nodules was evident in early images with a score 1-3. Highest specificity (91%) was obtained when the negative was defined as an absence of uptake-increase, in the late images. This study confirms that the most valuable feature of MIBI is the high NPV. Thus, with the appropriate interpretation method, high sensitivity and specificity, and moderate PPV can be obtained.

## INTRODUCTION

In Germany, more than 15 million people have thyroid nodules and about 90,000-100,000 patients undergo thyroid surgery each year; yet only approximately 6000 of these patients are diagnosed with thyroid cancer. This means that not even 1 in 1000 thyroid nodules is a malignant one [1-4]. To avoid such overtreatment, it is very important to improve the diagnostic approach to thyroid nodules.

Nodules with a diameter of ≥ 1 cm require a thyroid scintigraphy to evaluate the functional activity [5]. There is an increased suspicion of malignancy (2-5%) in hypo- and isofunctional nodules, and in these cases, further evaluation is needed [6].

[^99m^Tc]Tc-Sestamibi(MIBI) is increasingly used in practice for the investigation of thyroid nodules. The tracer accumulates in mitochondria-rich cells, common in hyperplasia, malignant tumors, or parathyroid adenomas [6]. This may help distinguish between benign and malignant nodules, which is important for the selection those patients who would benefit from surgery [7].

Fine needle aspiration biopsy (FNAB) is a safe and minimally invasive method for the evaluation of thyroid nodules [8]. However, FNAB can be problematic, especially in patients with multinodular goiter or in those with difficult to access lesions. Furthermore, this technique is highly examiner-dependent and the rate of non-diagnostic results varies between 2-32%, requiring a re-biopsy [9-15].

Previous retrospective studies showed that MIBI-imaging has a very high negative predictive value (NPV; mean > 97%, range 84-100%) [16-31]. However, most of the studies have a limited number of patients (range 25-83) [9, 16-19, 21, 23-31]. The largest study, with 130 patients, is from the Mexican study group of Hurtado-Lopez et al., which found that the NPV of MIBI-scintigraphy is 100% [20].

Another issue is that there is no standardized MIBI-imaging protocol; there can be either a single-phase protocol with late images (1-2 h post injection) or a dual-phase protocol with early (15-30 min post injection) and late images. The imaging can include planar images and/or single-photon emission computed tomography (SPECT) images [6].

The aim of this study was to examine the accuracy of MIBI-scintigraphy for the differentiation of thyroid nodules in the German population. The secondary objective was to determine the best acquisition time for the images. To our knowledge, this is the largest study, to date, evaluating the utility of MIBI in thyroid nodules.

## RESULTS

### Patients and thyroid nodules

The mean age of the 122 female (76%) and 39 male (24%) participants was 51 + 14 years (range 18-82 years). The mean volume of the nodules was 5.2 + 6.4 ml (range 0.1–37.6 ml). 33 patients had a single thyroid nodule and 128 patients had a multinodular goiter. In the technetium-99m-pertechnetate (TPT)-scintigraphy, 145 (90%) of the nodules were hypofunctional and 16 (10%) were indifferent. Characteristics of the thyroid nodules are summarized in Table 1.

**Table 1:** Characteristics of the thyroid nodules

Characteristic	Data (%)
Goiter	
Uninodular	33 (20.5%)
Multinodular	128 (79.5%)
Prior radioiodine treatment (RAI)	
Yes	11 (6.8%)
No	150 (93.2%)
Toxic nodular goiter	
Yes	25 (15.5%)
No	136 (84.5%)
Nodule functionality in [^99m^Tc]Tc-pertechnetate (TPT) scintigraphy	
Hypofunctional	145 (90.1%)
Indifferent	16 (9.9%)
Histopathological diagnosis	
Benign nodule	139 (86.3%)
Malignant nodule	22 (13.7%)

### Histopathological results

The histopathological examination revealed 139 (86%) benign and 22 (14%) malignant nodules. From the malignant tumors, 15 (68%) were papillary, four (18%) were follicular, and three (14%) were papillary cancer with follicular differentiation. There were no cases of anaplastic cancer. Medullary carcinomas were not observed, because all cases of elevated tumor marker calcitonin underwent a calcium stimulation test for further evaluation and did not receive a MIBI-scintigraphy.

### Diagnostic utility of fine needle aspiration biopsy (FNAB)

109 patients underwent a FNAB, 52 cytology cases (48%) were benign, 23 (21%) suspect, three (3%) were malignant neoplasm, and 31 (28%) were non-diagnostic (Table 2).

**Table 2:** Utility of fine needle aspiration biopsy (FNAB) for the differentiation between benign and malignant thyroid nodules

Cytology results of the FNAB	Histopathology results Data (%)		Total (%)
	Benign	Malignant	
Not done	45 (32.4%)	7 (31.8%)	52 (32.3%)
Normal	48 (34.5%)	4 (18.2%)	52 (32.3%)
Pathologic	1 (0.7%)	2 (9.1%)	3 (1.9%)
Intermediate/suspected	19 (13.7%)	4 (18.2%)	23 (14.3%)
Insufficient	26 (18.7%)	5 (22.7%)	31 (19.2%)
Total (%)	139 (100%)	22 (100%)	161 (100%)

Differentiation between benign and malignant tumors was not statistically significant, p = 0.058.

From the histopathologically benign nodules, 48 had normal cytology results and one case had pathologic cytology. There were 19 patients with intermediate cytology, meaning that the results were suspicious but unconducive, for example cellular atypia or follicular neoplasia. In 26 cases there was inadequate aspiration material.

From the malignant nodules, four had normal results, four had intermediate/suspect results, and two had pathological results in the cytology. Five aspirates were non-diagnostic.

The obtained p-value of the utility of FNAB to differentiate between benign and malignant nodules was 0.058 (not significant). Statistical analyses showed a good NPV of 92%, the PPV was 23%. Sensitivity and specificity were 60% and 71%, respectively (Table 3).

**Table 3:** Diagnostic values of fine needle aspiration biopsy (FNAB)

		Patients with thyroid nodules		
		Malignant nodules	Benign nodules	
**FNAB findings**	Positive (malignant)	**True positive**	**False positive**	PPV
		6 (7.7%)	20 (25.7%)	23%
	Negative (benign)	**False negative**	**True negative**	NPV
		4 (5.1%)	48 (61.5%)	92%
		SEN	SPE	
		60%	71%	

SEN = sensitivity, SPE = specifity, PPV = positive predictive value, NPV = negative predictive value.

### Imaging findings

**Figure 1:**
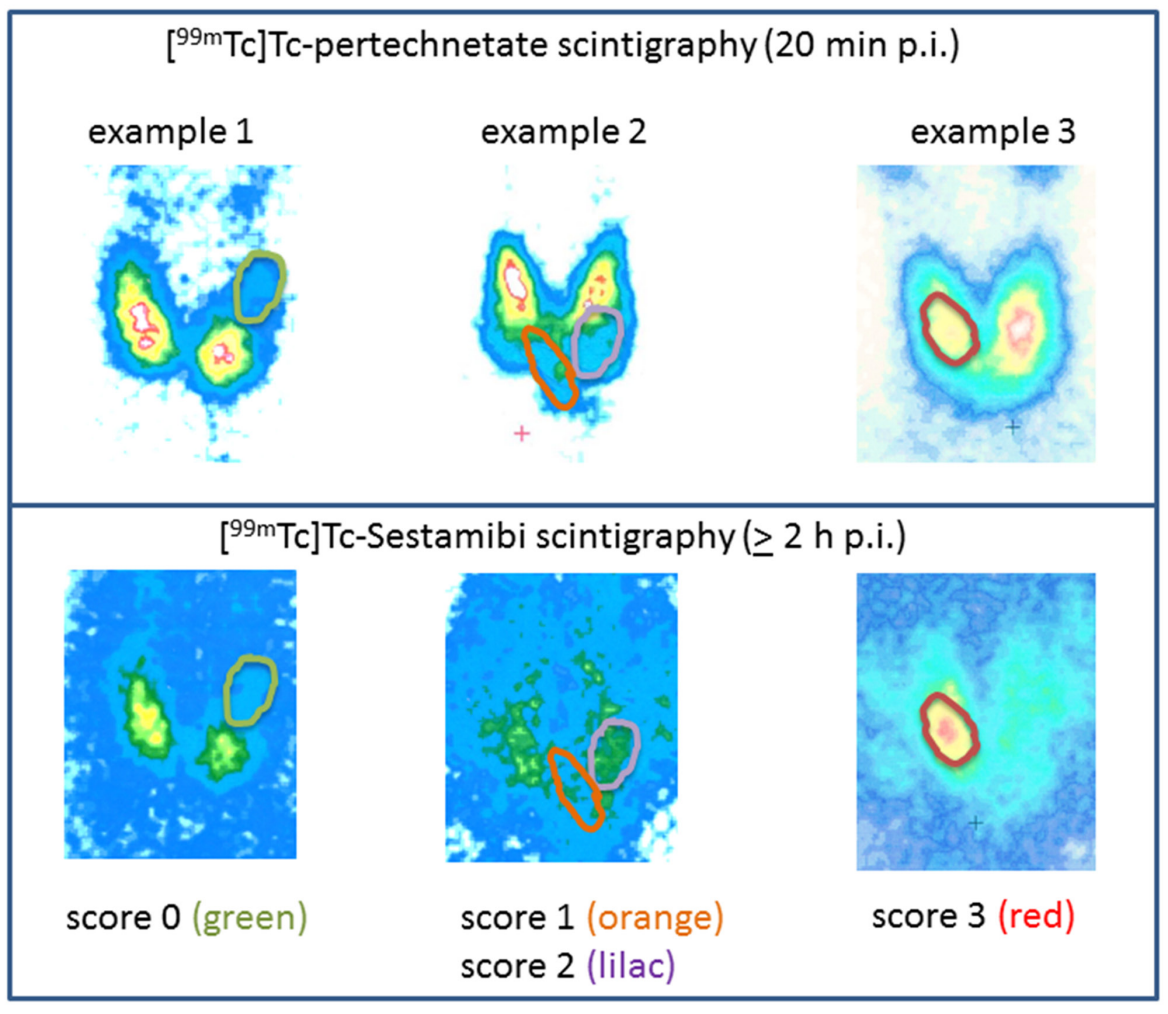
Examples of the scoring system of [^99m^Tc]Tc-Sestamibi (MIBI)- images The accumulation of the tracer in the thyroid nodule was classified as absent (score 0), low (score 1), isointense (score 2), or increased (score 3).

**Figure 2:**
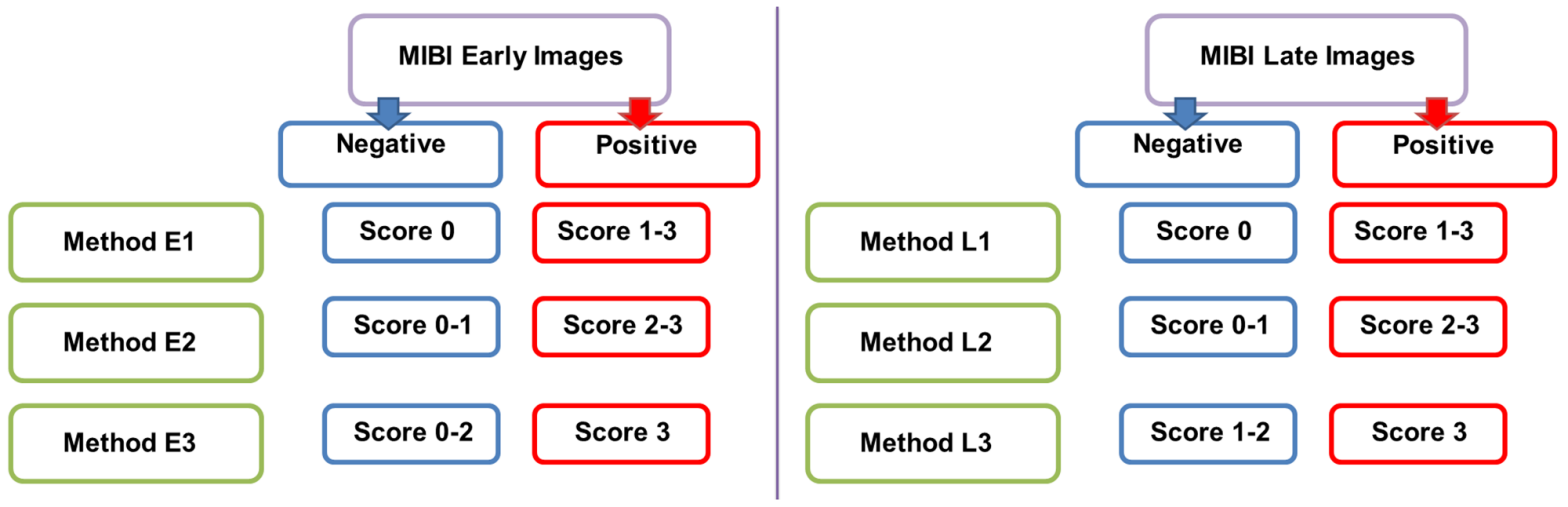
The different methods for the interpretation of early and late images of [^99m^Tc]Tc-Sestamibi (MIBI) scintigraphy Method E1: negative = score 0, positive = score 1-3. Method E2: negative = score 0-1, positive = score 2-3. Method E3: negative = score 0-2, positive = score 3. Method L1: negative = score 0, positive = score 1-3. Method L2: negative = score 0-1, positive = score 2-3. Method L3: negative = score 0-2, positive = score 3.

**Figure 3:**
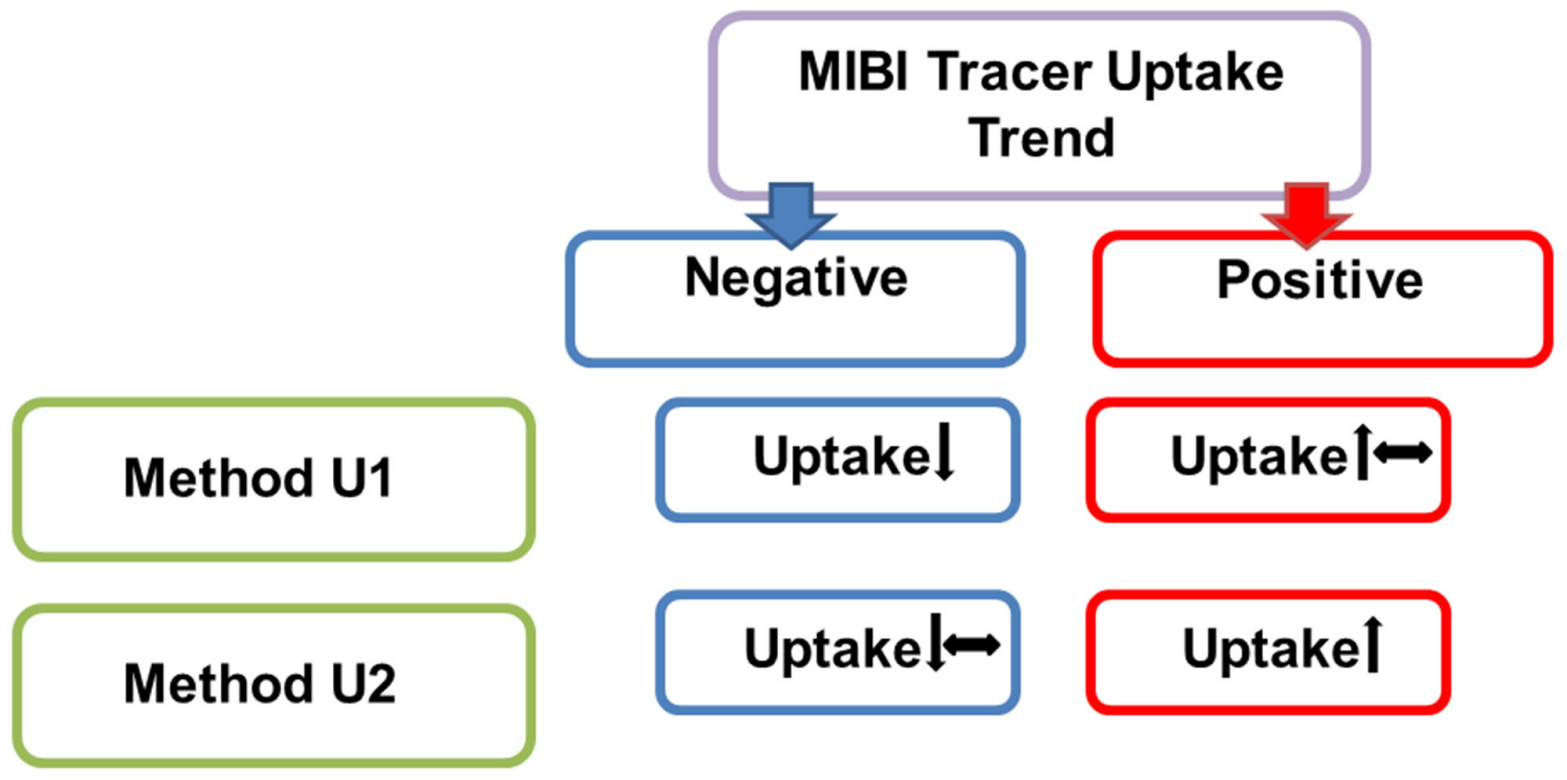
The different methods for the interpretation of tracer uptake trend of [^99m^Tc]Tc-Sestamibi (MIBI) scintigraphy in late images, compared to early images Method U1: negative = decreased uptake, positive = increased or constant uptake. Method U2: negative = decreased or constant uptake, positive = increased uptake.

**Figure 4:**
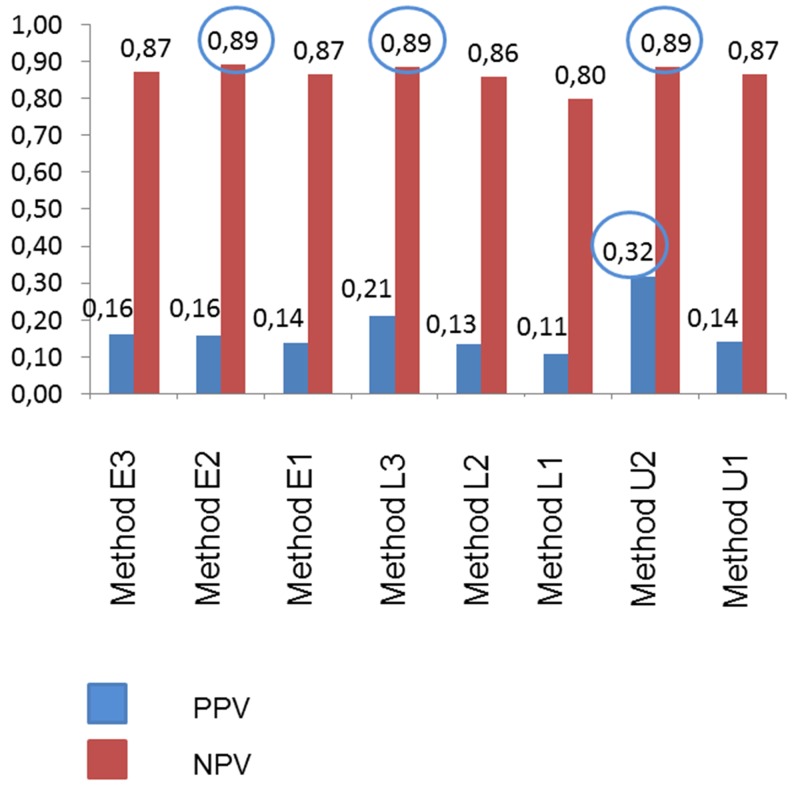
Positive predictive value (PPV) and negative predictive value (NPV) of different [^99m^Tc]Tc-Sestamibi (MIBI) imaging interpretation methods Early Images: Method E1: negative = score 0, positive = score 1-3. Method E2: negative = score 0-1, positive = score 2-3. Method E3: negative = score 0-2, positive = score 3. Late Images: Method L1: negative = score 0, positive = score 1-3. Method L2: negative = score 0-1, positive = score 2-3. Method L3: negative = score 0-2, positive = score 3. Interpretation of the tracer uptake trend in late images: Method U1: negative = decreased uptake, positive = increased or constant uptake. Method U2: negative = decreased or constant uptake, positive = increased uptake.

Table 4 presents the utility of MIBI-scintigraphy to differentiate between benign and malignant nodules. Flow charts of different imaging interpretation methods are depicted in Figures 1-3. Tables 5-7 show the diagnostic values (predictivity, sensitivity, specificity, and accuracy) of the different imaging interpretation methods. PPV and NPV of early and late images are also shown in Figure 4. Highest sensitivity (91%) for the detection of benign nodules was observed in the early images, when only score 0 findings were defined as negative. The highest specificity (91%) was obtained in the interpretation of the trend of the tracer uptake in late images; normal finding = decreased or constant uptake, abnormal finding = increased uptake.

**Table 4:** Utility of [^99m^Tc]Tc-Sestamibi (MIBI) for the differentiation between benign and malignant thyroid nodules

Method	Histopathology results Data (%)		Total (%)
	Benign	Malignant	
**Early images**			
Score 0	13 (9.4%)	2 (9.1%)	15 (9.3%)
Score 1	46 (33.1%)	5 (22.7%)	51 (31.7%)
Score 2	43 (30.9%)	8 (36.4%)	51 (31.7%)
Score 3	37 (26.6%)	7 (31.8%)	44 (27.3%)
Total (%)	139 (100%)	22 (100%)	161 (100%)
**Late images**			
Score 0	39 (28.1%)	10 (45.5%)	49 (30.4%)
Score 1	41 (29.5%)	3 (13.6%)	30 (18.6%)
Score 2	29 (20.9%)	1 (4.5%)	30 (18.6%)
Score 3	30 (21.6%)	8 (36.4%)	38 (23.6%)
Total (%)	139 (100%)	22 (100%)	161 (100%)
**Tracer uptake trend**			
Decrease (washout)	13 (9.4%)	6 (27.3%)	19 (11.8%)
Increase (retention)	78 (56.1%)	12 (54.5%)	90 (55.9%)
Constant (persistence)	52 (32.3%)	4 (18.2%)	48 (34.5%)
Total (%)	139 (100%)	22 (100%)	161 (100.0%)

The p-value from the early images was not significant, the late images achieved p = 0.044, and the washout/tracer retention method was p = 0.034.

**Table 5:** Diagnostic values of the early [^99m^Tc]Tc-Sestamibi (MIBI) imaging

Early images										
Variables	TP	TN	FP	FN	TOT	SEN	SPE	PPV	NPV	ACC
Imaging Interpretation	(%)	(%)	(%)	(%)	(%)					
**Method E1**	20	12	126	2	161	**0.91**	0.09	0.14	0.87	0.20
	(12.4)	(8.1)	(78.3)	(1.2)	(100)					
**Method E2**	15	59	80	7	161	0.68	0.42	**0.16**	**0.89**	0.46
	(9.3)	(36.6)	(49.7)	(4.3)	(100)					
**Method E3**	7	102	37	15	161	0.32	**0.73**	**0.16**	0.87	**0.68**
	(4.3)	(63.4)	(23)	(9.3)	(100)					

Method E1: negative = score 0, positive = score 1-3.

Method E2: negative = score 0-1, positive = score 2-3.

Method E3: negative = score 0-2, positive = score 3.

TP = true positive, TN = true negative, FP = false positive, FN = false negative, TOT = total of valid cases, SEN = sensitivity, SPE = specificity, PPV = positive predictive value, NPV = negative predictive value, ACC = accuracy.

Bold values highlight the most important results.

**Table 6:** Diagnostic values of the late imaging interpretation of [^99m^Tc]Tc-Sestamibi (MIBI) scintigraphy

Late images										
Variables	TP	TN	FP	FN	TOT	SEN	SPE	PPV	NPV	ACC
**Imaging Interpretation**	(%)	(%)	(%)	(%)	(%)					
**Method L1**	12	40	99	10	161	**0.55**	0.29	0.11	0.80	0.32
	(7.5)	(24.8)	(61.5)	(6.2)	(100)					
**Method L2**	9	80	59	13	161	0.41	0.58	0.13	0.86	0.55
	(5.6)	(49.7)	(36.6)	(8.1)	(100)					
**Method L3**	8	109	30	14	161	0.36	**0.78**	**0.21**	**0.89**	**0.73**
	(5.6)	(49.7)	(36.6)	(8.1)	(100)					

Method L1: negative = score 0, positive = score 1-3.

Method L2: negative = score 0-1, positive = score 2-3.

Method L3: negative = score 0-2, positive = score 3.

TP = true positive, TN = true negative, FP = false positive, FN = false negative, TOT = total of valid cases, SEN = sensitivity, SPE = specificity, PPV = positive predictive value, NPV = negative predictive value, ACC = accuracy.

Bold values highlight the most important results.

**Table 7:** Diagnostic values of the interpretation of the tracer uptake trend in late images from [^99m^Tc]Tc-Sestamibi (MIBI) scintigraphy

Tracer uptake trend										
Variables	TP	TN	FP	FN	TOT	SEN	SPE	PPV	NPV	ACC
Imaging Interpretation	(%)	(%)	(%)	(%)	(%)					
**Method U1**	10	78	61	12	161	**0.45**	0.56	0.14	0.87	0.55
	(6.2)	(48.4)	(37.9)	(7.5)	(100)					
**Method U2**	6	126	13	16	161	0.27	**0.91**	**0.32**	**0.89**	**0.82**
	(3.7)	(78.3)	(8.1)	(9.9)	(100)					

Method U1: negative = decreased uptake, positive = increased or constant uptake.

Method U2: negative = decreased or constant uptake, positive = increased uptake.

TP = true positive, TN = true negative, FP = false positive, FN = false negative, TOT = total of valid cases, SEN = sensitivity, SPE = specificity, PPV = positive predictive value, NPV = negative predictive value, ACC = accuracy.

Bold values highlight the most important results.

### Early images

Among the benign nodules the score was 0 in 13 nodules, while 46 nodules had a low MIBI-uptake (score 1), in comparison to the paranodular tissue. Another 43 nodules had a score 2, and 37 lesions had score 3.

Among the malignant nodules, the score was 0 in two nodules. Five lesions had a score 1, eight were isointense (score 2), and seven nodules showed increased uptake (score 3) in the scintigraphy.

The ability of the early images to differentiate between benign and malignant nodules was not significant.

### Late images

In the late images, among the benign nodules, 39 had no uptake (score 0), 41 had a score 1, and 29 had a score 2. 30 benign lesions had a score 3.

Among the malignant nodules, the score in the late images was 0 in 10 lesions, 1 in three nodules, and 2 in one nodule. Eight nodules showed high uptake (score 3).

In summary, the late images could reliably differentiate between benign and malignant nodules (p = 0.044, according to chi-square-test).

### Washout/tracer retention in the late images

Among the benign lesions, tracer uptake in the late images, compared with the early images, remained constant in 48 cases. There was an uptake decline in 78 of the lesions and an uptake increase in 13 lesions.

Among the malignant nodules, there was a sufficient washout in 12 cases, in four nodules the uptake was constant, and six nodules showed an increased retention of the tracer over time.

The washout/tracer retention method showed good reliability (p = 0.034) in terms of differentiation between benign and malignant nodules.

## DISCUSSION

There are several publications reporting the diagnostic value of MIBI-scintigraphy for the evaluation of thyroid nodules. Most impressing is MIBI’s high negative predictive value, of up to 100%; mean > 97% [9, 16-31]. Only Kresnik et. al. reported a much lower NPV of 84%; however, the study included only 62 patients [21]. Most of the studies have a small patient cohort (range 25-83 patients) [9, 16-20, 22-31]. The largest MIBI study (130 patients) from Hurtado Lopez et. al. was conducted in Mexico, where there is a high prevalence (38%) of thyroid carcinomas in this patient population [6, 20]. In comparison, the prevalence in Germany is only 11% [6]. It is the opinion of the authors, that image interpretation should be adapted to the prevalence of thyroid carcinomas in the studied population.

In the current study, the best NPV (89%) of thyroid nodules was observed in lesions with a score of 0-1 in the early images, and a score of 0-2 and in the late images (86% NPV). Good NPV (89%) was also obtained in the interpretation of the trend of the uptake over time; negative finding = decreased or constant uptake in the late images. This method also showed the best specificity of 91%. The reliable NPV of MIBI-scintigraphy can justify a “wait-and-see” strategy.

Another consideration is the positive predictive value of MIBI-scintigraphy. The PPV varies in the published literature between < 10% and > 61% [9, 16-31]. The utility of this value can be relevant for the indication for surgery. Our data show that the best PPV was obtained in the interpretation of tracer uptake trend. If a lesion has an increasing uptake in the late images, the PPV is 32%, and this can justify surgery.

False-negative findings are rarely reported in the literature. Examples are two patients with undifferentiated / anaplastic carcinoma from the study of Kresnik et al and Foldes et al [19, 21]. However, there were also small number of patients with differentiated cancer (papillary n=3 / follicular n=1), who had a negative MIBI-scan [26, 28, 29]. In our patient group, there were two cases of score 0 malignant nodules in the early images, and 10 false negative results in the late images. The false negatives may indicate that a continuous follow-up with ultrasound of the neck and if necessary repeated FNAB are needed in these patients.

We used the dual-phase study protocol with planar images for performing MIBI. The late images could reliably differentiate between benign and malignant nodules, p = 0.044. Good reliability for the differentiation (p = 0.034) was also revealed in the interpretation of the tracer uptake trend (washout or tracer retention) in the late images. However, better diagnostic values could have been achieved with SPECT imaging. For example, the study of Schenke et. al. compared SPECT with planar MIBI-imaging, achieving a much better NPV; 100% versus 91.7% [27].

There are some limitations and biases in the current study which need to be mentioned. Firstly, not all patients underwent a FNAB. From the 109 patients who had an ultrasound-guided FNAB, 31 (28%) had non-diagnostic biopsies. A better pre-operative diagnostic procedure would have decreased the benign nodules in this study. Secondly, we informed patients who did not undergo a FNAB, or who had an inadequate biopsy, or who had a FNAB with no evidence of malignancy, that the only way to exclude a malignancy is to undergo surgery. This may have raised the number of cases of operated patients. Thirdly, the majority of the malignant nodules were papillary carcinoma (68%); therefore, it is uncertain if the utility of MIBI-scintigraphy is the same for all thyroid cancer entities. Furthermore, there were no cases of anaplastic cancer in this study. This may be because anaplastic cancer is often clinically classified as suspect (fast growing and irregular in the ultrasound) and directly referred for surgical evaluation. Finally, there were patients who received MIBI-scintigraphy despite suspicious FNAB findings. The reason for this is that these patients received both a FNAB and MIBI on the same day, and we were not aware of the cytopathology results. To avoid such bias in the future, and to confirm the reliability of MIBI, prospective studies are needed.

In conclusion, the current study confirms that the most valuable feature of MIBI-scintigraphy is its NPV. Using a dual-phase protocol, 89% NPV could be achieved either in early images of lesions with a score 0-1, or in late images with a score 0-2. Furthermore, with the appropriate image interpretation method, a high sensitivity (91%), high specificity (91%) and moderate PPV (32%) can be obtained. The current study confirms that the most valuable feature of MIBI is the NPV. Thus, with the appropriate image interpretation method, high sensitivity and specificity, and moderate PPV can be obtained.

## MATERIALS AND METHODS

### Patients

In total, 603 patients underwent a MIBI-scintigraphy at our department between October 2005 and August 2014. All patients had at least one thyroid nodule, which was cold/indifferent in the [^99m^Tc]Tc-pertechnetate (TPT)-scintigraphy. At the time this study was conducted, MIBI was not an established diagnostic tool. For that reason, we informed patients who did not undergo a FNAB, or who had an inadequate biopsy, or who underwent a FNAB with no evidence of malignancy, that the only way to exclude a malignancy is surgery. Patients with elevated tumor marker calcitonin and suspicion of medullary cancer did not receive a MIBI-scintigraphy. In these cases we used the calcium stimulation test for further evaluation. From the 603 studied patients, 161 received a histopathological diagnosis after thyroid surgery, and were included for further analysis. The local ethics committee of our university approved this retrospective study; informed consent was obtained from all participants.

### Imaging and interpretation

All patients underwent TPT-scintigraphy followed by MIBI-scintigraphy. After intravenous injection of MIBI (standard dose 370 MBq) we used a dual-phase protocol with planar images approximately 20 min post-injection (p.i.) and 2 h p.i.

The uptake in the examined nodule was compared with the paranodular thyroid tissue. The findings were classified visually; accumulation of the tracer in the thyroid nodule was classified as absent (score 0), low (score 1), isointense (score 2), or increased (score 3). Examples of this scoring system are presented in Figure 1. We also considered the washout or the tracer-retention in the late images, compared to the early images.

A correlation with the TPT-scintigraphy showed either a “match” (i.e., concordant decreased uptake of both tracers) or a “mismatch” (i.e., cold nodule with increased MIBI-uptake).

### Fine needle aspiration biopsy (FNAB)

109 patients underwent a FNAB. The biopsy was performed through guided ultrasound using a 20-gauge needle attached to a 20 ml Cameco syringe-pistol. Smears were made and air-dried slides were stained with hematoxylin-eosin. The remaining aspirate material in the syringe was rinsed with 0.9% sodium chloride. The fluid material was centrifuged, the sediment was smeared and slides were prepared. Adequacy of the aspirates was assessed on the basis of the guidelines of the Papanicolaou Society [32].

### Histopathology

The gold standard for histologic diagnosis served as a reference. From the 603 patients, 161 patients underwent surgery for further histopathological examination of the thyroid nodules. Routine staining with hematoxylin-eosin, Elastica van-Giesson and immunochemistry, if necessary, was performed.

### Statistical analyses

Statistical analyses were performed with IBM SPSS software version 22. Different image interpretation methods were compared for their utility to differentiate between benign and malignant nodules. We used the chi-square test to determine the statistical significance of the results. The significance level was accepted at 5%. Additionally, to obtain the validity of the imaging, we classified the findings into true positive (TP), true negative (TN), false positive (FP), and false negative (FN). Sensitivity (SEN) was calculated as TP/(TP + FN), specificity (SPE) as TN/(TN + FP), and accuracy (ACC) as (TP + TN)/(TP + TN + FP + FN). The positive predictive value (PPV) was defined as TP/(TP + FP), the negative predictive value (NPV) as TN/(TN + FN).
